# Intraoperative pyloric drainage is unnecessary during esophagectomies: a meta-analysis and systematic review of randomized controlled trials

**DOI:** 10.3389/pore.2024.1611823

**Published:** 2024-08-06

**Authors:** Armand Csontos, Dávid Németh, Lajos Szakó, Gergő Berke, Dóra Lili Sindler, Dávid Berki, Csenge Papp, Péter Hegyi, András Vereczkei, András Papp

**Affiliations:** ^1^ Department of Surgery, Clinical Center, Medical School, University of Pécs, Pécs, Hungary; ^2^ Institute for Translational Medicine, Medical School, University of Pécs, Pécs, Hungary; ^3^ Institute of Bioanalysis, Medical School, University of Pécs, Pécs, Hungary; ^4^ János Szentágothai Research Centre, Medical School, University of Pécs, Pécs, Hungary; ^5^ Department of Emergency Medicine, Medical School, University of Pécs, Pécs, Hungary; ^6^ First Department of Surgery, Military Hospital Medical Centre, Hungarian Defense Forces, Budapest, Hungary; ^7^ First Department of Medicine, Medical School, University of Szeged, Szeged, Hungary; ^8^ Hungary Centre for Translational Medicine, Semmelweis University, Budapest, Hungary; ^9^ Division of Pancreatic Diseases, Heart and Vascular Center, Semmelweis University, Budapest, Hungary

**Keywords:** esophageal cancer, esophagectomy, esophageal surgery, pyloroplasty, minimal invasive

## Abstract

**Objective:** The topic of this meta-analysis is the comparison of gastric conduit esophageal reconstructions with or without pyloroplasty.

**Background:** Surgical procedures, especially minimal invasive esophagectomy (MIE) can be a curative treatment in the early stages of esophageal cancer. Previously, intraoperative pyloroplasty was routinely performed, but nowadays it became debated again in the light of minimally invasive esophagectomy.

**Methods:** A comprehensive search was performed in multiple databases to identify randomized controlled trials investigating the topic. Two independent authors performed the selection based on predefined criteria. Statistical analysis was performed to assess any significant difference, then the bias and quality of the data were estimated.

**Results:** Nine relevant RCTs consisting of 529 patients with esophageal cancer were identified. No significance was found in mortality [odds ratio (OR): 0.85; *p* = 0.642], anastomosis leakage (OR: 0.57; *p* = 0.254), respiratory morbidity (OR: 0.51; *p* = 0.214) and vomiting (OR: 0.74; *p* = 0.520), however the results about gastric emptying time (GET) were controversial (weighted mean difference (WMD): −67.71; *p* = 0.009, OR: 2.75; *p* = 0.072). Significant heterogeneity was not detected except for GET. Trial sequential analyses (TSA) show that a certain conclusion would require more data except in the binary variables of GET.

**Conclusion:** We conclude that the pyloric drainage procedure is not routinely necessary, but further well-designed studies would be needed, especially in Europe.

## Introduction

Esophageal cancer is the 8th most common malignancy in the world with more than 600,000 cases (3.1% of all) and it is responsible for more than 500,000 deaths (5.5% of all) on a yearly basis, thus being the 6th most common cause of cancer mortality. The worldwide prevalence in the 1-, 3-, and 5-year-periods are 2.4%, 1.6%, and 1.3%, respectively [1].

The two histological subtypes are squamous cell cancer (SCC) and adenocarcinoma (AC). In 2012 the incidences were estimated to be more than 398,000 (5.2 per 100,000 people) for SCC and 52,000 (0.7 per 100,000 people) for AC globally. Although SCC is still the leading histological form, the number of AC in the Western world is gradually increasing, including Hungary [2]. Esophageal cancer is more common among men, the male-to-female ratios being 2.7 and 4.4 in SCC and AC, respectively [3].

Nowadays esophagus carcinoma still has a poor 5-year survival rate, which is estimated at around 19% in the United States and 12% in Europe [4, 5]. According to tumors, under 3 cm, in stage I–III, the 5-year survival rate can be estimated at 86%–22%. However, in the case of a larger tumor the prognosis is significantly worse, around 27%–8% [6]. As presented, esophageal cancer itself is accompanied by significant mortality and morbidity, to which the possibility of early metastases also contributes. Therefore surgical treatment may also show poor outcomes which depend on the stage of the tumor, the condition of the patients, and the skill of the surgeon [7].

To treat esophageal cancer, the 8th edition of the UICC-AJCC TNM Classification recommends esophagectomy in stages I-IIB, when the operation can be a curative treatment, especially with minimal invasive esophagectomy (MIE) or a robot assisted minimal invasive esophagectomy (RAMIE), both are becoming the gold standard procedures [8, 9]. Performing intraoperative pyloric drainage has long been considered an integral part of the elective esophagectomy, but its application along minimally invasive resections, lead to technical difficulties and prolonged operation time, therefore the necessity of pyloric drainage procedures became debated again [10]. The importance of the topic is also supported by the fact, that new articles are published nowadays, but the literature is still controversial [11–13].

Before the spread of minimal invasive techniques, performing pyloroplasty or pyloromyotomy during esophagectomy was recommended with the aim of reducing gastric stasis, furthermore, providing a better quality of life [14]. From this aspect pyloric drainage can prevent anastomosis leakage, and postoperative pulmonary complications, which can lead to shorter hospital stays, and a lower risk of overall perioperative mortality [15].

Contrary to this, other authors demonstrated that the long-term complications of pyloric interventions facilitate biliary reflux and reflux esophagitis, which can lead to poor quality of life [16]. Therefore, pyloric drainage should be avoided as it is ineffective on the gastric emptying time and may also cause biliary reflux esophagitis [17].

Several papers describe, that pyloroplasty has no benefit in terms of mortality or any sort- and long-term complications, however, the effects of pyloroplasty on the relative risk of delayed gastric emptying is still controversial [16, 18, 19].

Our aim was to investigate whether pyloric drainage procedures are advantageous compared to the omission of these interventions in terms of mortality, gastric emptying time, anastomosis leakage, vomiting, and aspiration pneumonia by performing a meta-analysis according to the latest methodologies. Only the highest quality randomized controlled trials were selected for the most accurate and reliable results.

## Methods

We registered our protocol to the medRxiv server in advance, under the number 10.1101/2022.08.24.22279164 [20].

### Search strategy

We included studies, which reported on patients treated with esophagectomy due to esophageal cancer. We excluded patients with esophageal resections due to any other causes. The investigational group of our analysis consisted of those with any kind of intraoperative pylorus drainage procedure, while those without it formed the control group. The investigated outcomes were mortality, gastric emptying time (GET), anastomosis leakage, aspiration, vomiting, and respiratory complications. Although our trial was registered in 2022, an up-to-date systematic search was conducted on the 18th of March 2024 to renew our database, allowing us to find the latest RCTs. We searched the following databases: MEDLINE (via PubMed), Embase, Cochrane Library, Web of Science, and Scopus. We did not use any restrictions. The search phrase was defined as “[(esophagus OR oesophagus OR esophageal OR oesophageal) AND (surgery OR surgical OR operative OR operation OR resection)] OR (esophagectomy OR oesophagectomy OR minimal invasive OR MIE OR RAMIE) AND (pyloroplasty OR pyloromyotomy OR drain*).”

### Selection

The selection was performed by two independent authors (A. C. and L. S.) and the disagreements were resolved by a third author (A. P.). EndNote (EndNote X9.3.3, Alfasoft AB, Göteborg, Sweden) was used for the selection steps, which was done by the title, abstract and full-text. We included randomized clinical trials with esophageal cancer patients, who were treated by esophagectomy and pyloric drainage. The trial also had to contain a control group in which no pyloric drainage procedure was performed. References of included articles and former meta-analyses were screened for additional publications. The reasons for exclusion were the retrospective study design, lack of randomization or control group, pediatric trials or animal studies, non-malignant esophageal pathologies, additional surgical interventions, and postoperative pylorus drainage procedure (e.g., balloon dilatation, botulinum toxin injection) or peroral endoscopic myotomy (POEM).

### Data extraction

Two independent authors (A. C. and L. S.) extracted data from the articles based on pre-agreed criteria using an Excel data sheet (Office 365, Microsoft, Redmond, WA, United States) for collection and methodization. Data on publication, demography, pathology, operation, and investigated outcomes were extracted.

### Statistical analysis

The meta-analytic calculations were performed using the STATA statistical software package (StataCorp. 2017. Stata Statistical Software: Release 15. College Station, TX: StataCorp LLC). Recommendations of the working group of the Cochrane Collaborations were used during the data synthesis. From raw data, pooled odds ratios (ORs) with their 95% confidence intervals (CIs) in the case of dichotomous variables were calculated. In the case of continuous variables, weighted mean differences (WMD) with their 95% confidence intervals were calculated. The random effect model with the estimation of DerSimonian and Laird [21] was used, and the results were displayed on a forest plot. Cochrane’s Q and the I^2^ statistics were used to assess heterogeneity. Statistical significance was achieved in the case of *p* < 0.05. Trial sequential analysis was performed to assess the necessary number of cases to obtain conclusive evidence in each outcome using the trial sequential analysis tool from Copenhagen Trial Unit (Centre for Clinical Intervention Research, Denmark).

### Risk of bias assessment

Two independent authors (A. C. and L. S.) used the Risk of Bias Assessment Tool version 2 by Cochrane to assess the possible biases, and the disagreements were resolved by a third author (A. P.) [22].

### Certainty of evidence

To assess the certainty of evidence the GRADE approach was applied by two independent authors (A. C. and L. S.) and a third author (A. P.) to resolve the disagreements [23].

## Result

### Results of the selection process

In five databases 11,141 articles were identified. After the selection procedures eight articles were included [18, 24–30]. One additional article was found from the references of former meta-analysis [31]. In summary, nine relevant articles were included in the quantitative synthesis [18, 24–31].

We excluded four articles because of a missing control group, and in the case of three articles, full-text articles could not be obtained, even by contacting the authors [32–38].

**FIGURE 1:**
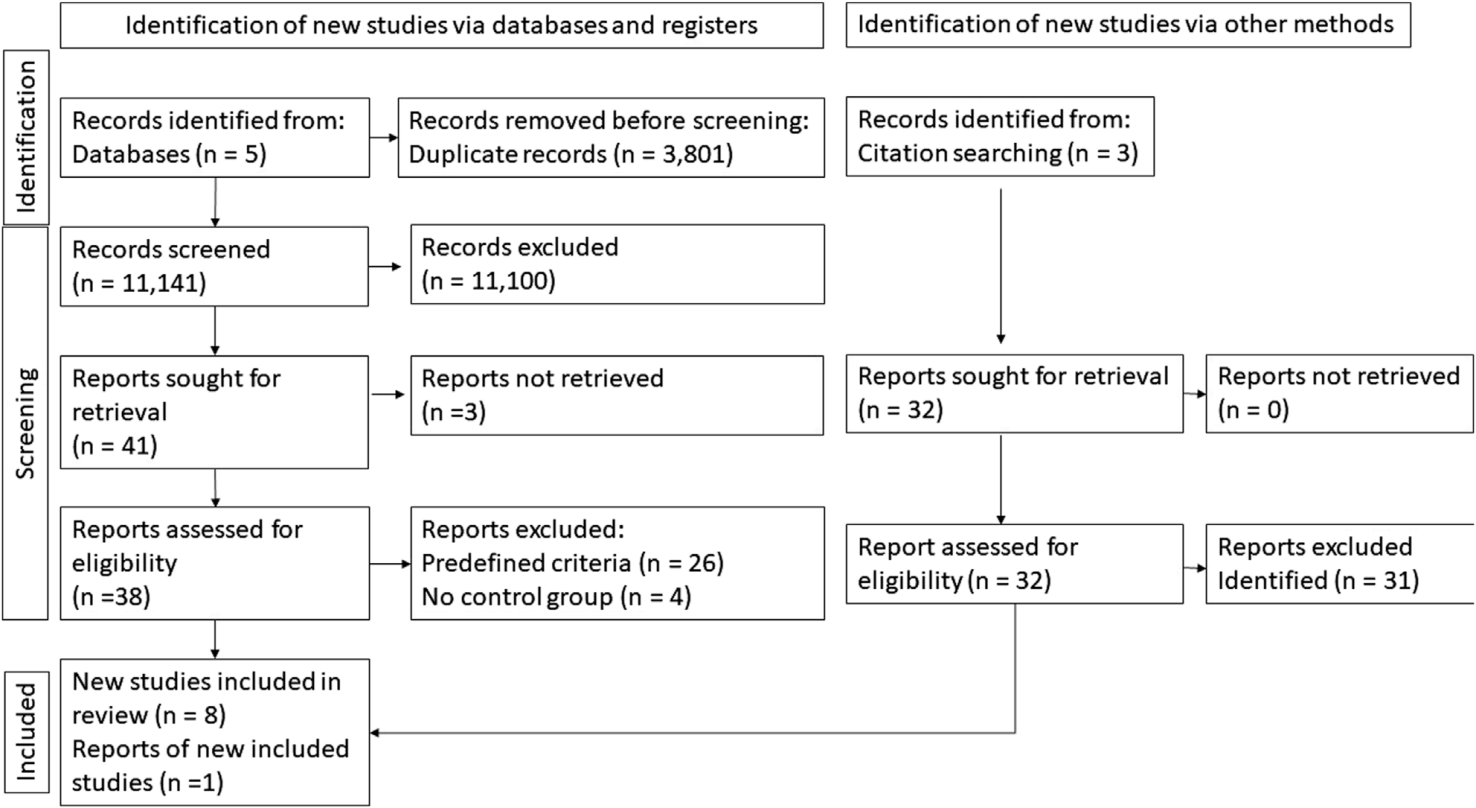
Selection flow chart.

The detailed results of the selection are presented below (Figure 1).

### Characteristics of the studies included

All these nine studies were randomized controlled trials published between 1985 at 2016. Table 1 shows the details of these studies.

**TABLE 1:** The characteristic of the study.

Author	Year of publication	Country	No. of patients	Man/Woman	Age	Type of pyloroplasty	Follow-up (mean)
Huang et al. [31]	1985	China	52	—	—	Heineke - Mikulicz	—
Gupta et al. [30]	1989	India	24	—	—	Heineke - Mikulicz	—
Mannel et al. [25]	1990	South Africa	40	—	53.0	Aust	5 MTH
Chattopadhyay et al. [26]	1991	India	24	—	—	Heineke - Mikulicz	—
Fok et al. [24]	1991	Hong Kong	200	9.5	61.0	Heineke - Mikulicz	17 MTH
Chattopadhyay et al. [27]	1993	India	24	3.8	49.0	Heineke - Mikulicz	6 MTH
Kao et al. [29]	1994	China	38	3.8	62.3	Pyloroplasty (n = 15) Pyloromyotomy (n = 4)	—
Zieren et al. [28]	1995	Germany	107	3.9	57.5	Pyloroplasty	6 MTH (median)
Mohajeri et al. [18]	2016	United Arab Emirates	20	2.3	57.4	Pyloroplasty	—

Table 1 is containing the character of the studies. No, number; n, number of patients who underwent intervention; MTH, month. An empty cell means no information is mentioned.

### Characteristics of the patients

The studied population consisted of 529 patients diagnosed with esophageal cancer, where malignant tumors were confirmed in 524 (99%) cases. 257 (48.6%) patients were randomly assigned to the intraoperative pyloric drainage group, where 253 (98.4%) pyloroplasties and 4 (1.6%) pyloromyotomies were performed. The weighted average age of the population was 58.6 years, and there were 6.7 times more men than women. The patients were in 20% European, 73% Asian, and 7% African. Data on follow-up were very heterogeneous. Patients were followed-up from 2 weeks to 15 years, but the average follow-up time was at 1 year.

### Mortality

**FIGURE 2:**
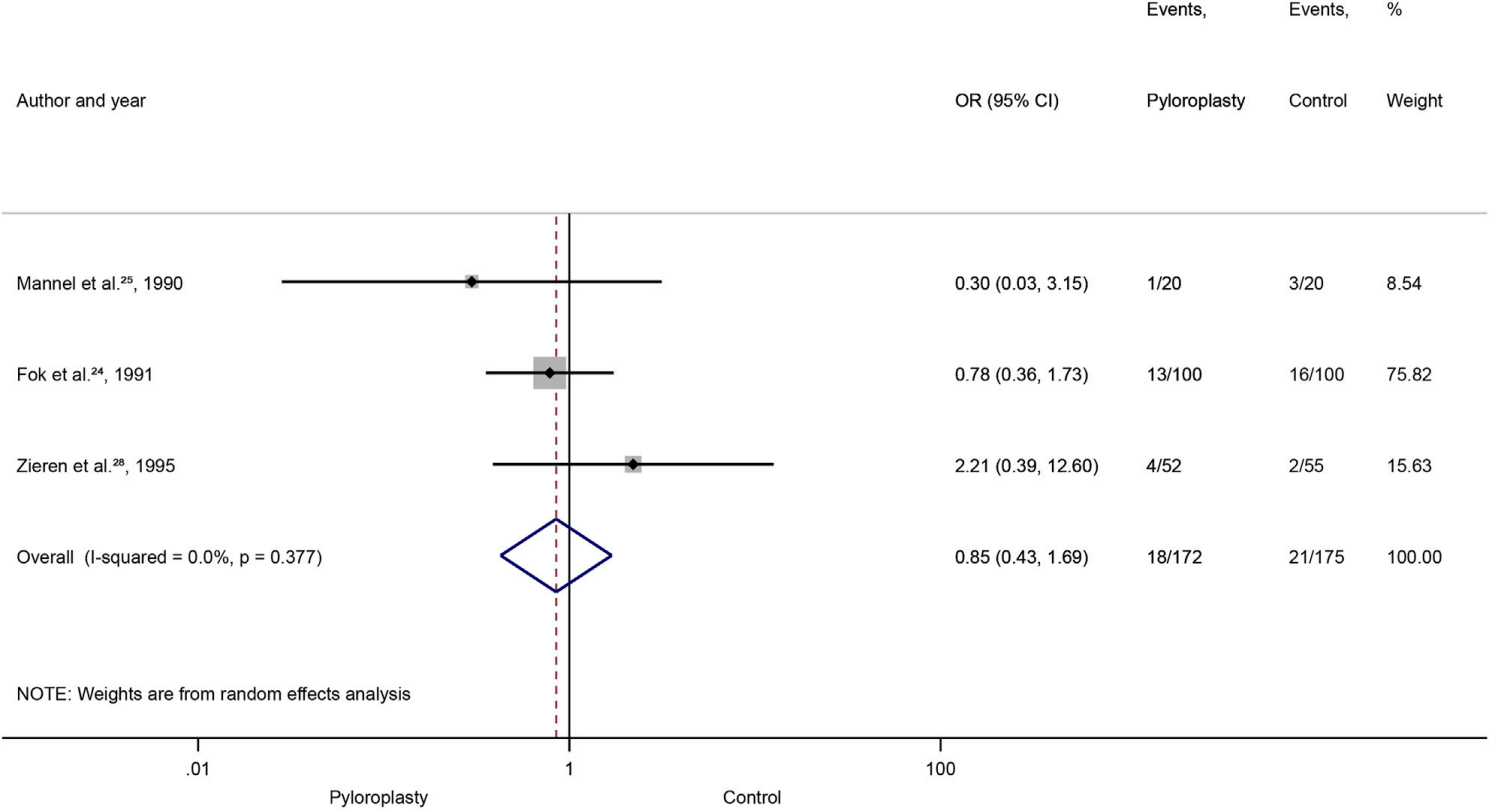
Mortality, Forrest plot, OR: odds ratio: CI: confidence interval.

We included 347 patients from three RCTs in the analysis [25, 28, 30]. No difference was found between the pyloroplasty and the control group (OR: 0.85, 95% CI: [0.43, 1.69], *p* = 0.642), and significant heterogeneity was not detected (I^2^ = 0%, *p* = 0.37) (Figure 2). The TSA analysis could not be performed as the insufficient difference between the two groups were detected.

### Anastomosis leakage

367 patients from four RCTs were included [18, 25, 28, 30], and no difference was found between the pyloroplasty and the control group (OR: 0.57, 95% CI: [0.21, 1.51], *p* = 0.254), and significant heterogeneity was not detected (I^2^ = 41%, *p* = 0.16), however, according to the TSA analysis 2006 patients would have been needed to conclude (Supplementary Figures S1, S2).

### Respiratory morbidity

We had 196 patients in three RCTs [25, 26, 30]. No difference was found between the pyloroplasty and the control group (OR: 0.51, 95% CI: [0.18, 1.48], *p* = 0.214), and significant heterogeneity was not detected (I^2^ = 0%, *p* = 0.47), however, 534 patients would have been required, based on the TSA analysis (Supplementary Figures S3, S4).

### Vomiting

The four included RCTs provided 316 patients [25, 27, 30, 31]. No difference was found between the pyloroplasty and the control group (OR: 0.74, 95% CI: [0.30, 1.84], *p* = 0.520), and significant heterogeneity was not detected (I^2^ = 0%, *p* = 0.62) however, 5,043 patients would have been required to make a conclusive statement (Supplementary Figures S5, S6).

### Gastric emptying time

**FIGURE 3:**
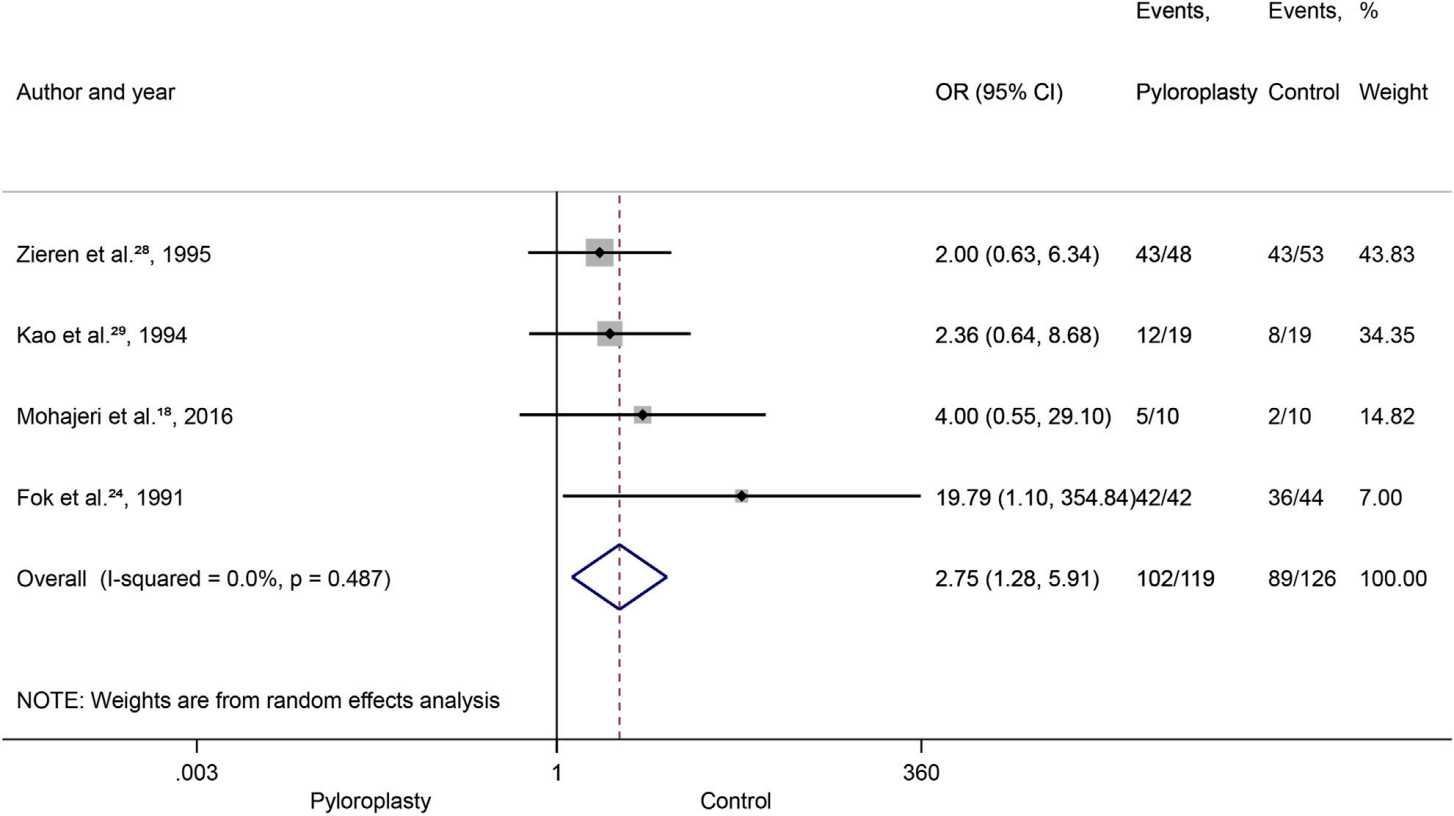
Gastric emptying time (GET) dichotomic data, Forrest plot, OR: odds ratio, CI: confidence interval.

We had 245 patients from four RCTs for dichotomous data [18, 28–30]. The results of the statistical analysis showed that patients in the control group had a significantly, 2.75-fold higher chance for the delayed GET than the pyloroplasty group (OR: 2.75, 95% CI: [1.28, 5.91], *p* = 0.009), and significant heterogeneity was not detected (I^2^ = 0%, *p* = 0.48) (Figure 3). According to the TSA analysis, the required number of patients was achieved (Supplementary Figure S7).

We had 253 patients from seven RCTs for continuous data [18, 24–26, 28, 29, 31]. No difference was found between the two groups (WMD: −67.71, 95% CI: [−141.60, 6.18], *p* = 0.072), and significant heterogeneity was detected (I^2^ = 99%, *p* < 0.001), however 623 patients would have been required for this outcome to be conclusive (Supplementary Figures S8, S9).

### Risk of bias

We used Risk of Bias Assessment Tool 2, which estimated all outcomes as low risk (Table 2) [22].

**TABLE 2:** The result of risk of bias tool 2.

Outcome	Article	Randomization process	Deviations from the intended interventions	Missing outcome data	Measurement of the outcome	Selection of the reported result	Overall
Mortality	Mannel et al. [25], 1990	+	+	+	+	+	+
	Fok et al. [24], 1991	+	+	+	+	+	+
	Zieren et al. [28], 1995	+	+	+	+	+	+
Anastomosis Leakage	Mannel et al. [25], 1990	+	+	+	+	+	+
	Fok et al. [24], 1991	+	+	+	+	+	+
	Zieren et al. [28], 1995	+	+	+	+	+	+
	Mohajeri et al. [18], 2016	+	+	+	+	+	+
Respiratory Morbidity	Mannel et al. [25], 1990	+	+	+	+	+	+
	Chattopadhyay et al. [26], 1991	+	+	+	+	+	+
	Zieren et al. [28], 1995	+	+	+	+	+	+
Vomiting	Huang et al. [31], 1985	+	+	+	+	+	+
	Mannel et al. [25], 1990	+	+	+	+	+	+
	Chattopadhyay et al. [26], 1991	+	+	+	+	+	+
	Zieren et al. [28], 1995	+	+	+	+	+	+
Gastric Emptying Time (dichotomic)	Fok et al. [24], 1991	+	+	+	+	+	+
	Kao et al. [29], 1994	+	+	+	+	+	+
	Zieren et al. [28], 1995	+	+	+	+	+	+
	Mohajeri et al. [18], 2016	+	+	+	+	+	+
Gastric Emptying Time (continuous)	Huang et al. [31], 1985	+	+	+	+	+	+
	Gupta et al. [30], 1989	+	+	+	+	+	+
	Mannel et al. [25], 1990	+	+	+	+	+	+
	Chattopadhyay et al. [26], 1991	+	+	+	+	+	+
	Fok et al. [24], 1991	+	+	+	+	+	+
	Kao et al. [29], 1994	+	+	+	+	+	+
	Mohajeri et al. [18], 2016	+	+	+	+	+	+

Table 2 is containing the results of Risk of Bias Tool 2, which resulted in low risk in all outcomes. +: Low risk; !: Some concerns; -: high risk.

### Certainty of evidence

The quality of the evidence (GRADE) of mortality, anastomosis leakage, respiratory morbidity, vomiting, and the dichotomic data of gastric emptying time, were estimated as moderate, due to older articles and the fact, that most of the studies were conducted in an Asian country. The continuous data of the gastric emptying time was low quality, therefore, it should be interpreted accordingly (Supplementary Table S1) [23].

## Discussion

Although the debate about the benefit of pyloroplasty is an old-school topic, new articles are still being written about the subject [11–13]. Pyloroplasty following esophagectomy in elective esophageal surgery is still recommended as a routine procedure by many authors, but recommendations apply for a modified version of the technique [11, 13, 39, 40]. In contrast, our investigation has demonstrated, that this step of esophagectomy has no benefits in the practice. In other words, its omission can significantly shorten the surgical time, which can be beneficial for the patient. This fact can be especially important in relation to minimal invasive techniques, which is supported by Nobel et al. with their retrospective study [12].

Minimally invasive esophagectomy (MIE) appears to be beneficial and is increasingly moving towards robot-assisted techniques and has become the gold standard procedure with widespread use [41]. These techniques have many advantages, and only come with negligible limitations. Siaw-Acheampong et al. compared laparoscopic, thoracoscopic, totally minimally invasive, and robotic esophagectomy and they found decreased perioperative morbidity and hospitalization period against open surgery, while MIE did not influence perioperative mortality. Szakó et al. also confirmed these findings in terms of pulmonary complications [41]. On the other hand, these procedures are associated with technical difficulties and prolonged operation time [10]. This is the reason why intraoperative pyloroplasty itself is an additional intervention which can logically extend the otherwise prolonged operation, which may even generate perioperative complications, therefore the debate about the necessity of pyloroplasty arose again.

Through technical developments new postoperative methods have emerged, such as balloon dilatation, pyloric bouginage, endoscopic myotomy, or botulinum toxin injection [11, 13, 18, 42]. Although all are safe and accessible, and can be performed instead of surgical pyloroplasty in some cases, there are still limitations and no evidence about routine usage. Per-oral gastric pyloromyotomy (GPOP), which was developed in the pattern of per-oral endoscopic myotomy (POEM), may be useful in case of resistance to balloon dilatation or botulinum toxin injection, however, it has limitations due to its technical difficulties [43]. In the case of botulinum toxin injection there are data about an increased chance of reoperation [44]. Despite their limitations, all these methods are available, safe, and quick, therefore potentially may replace intraoperative pyloroplasty. Based on these, our focus of interest is highlighted again [12, 32, 44].

Clear evidence is still missing, considering the best type of minimally invasive pyloric drainage procedure or whether pyloric drainage should be recommended during surgery or not [11, 12, 44].

Almost a decade ago, Gaur et al. and Khan et al. stated that the omission of pyloroplasty had no benefits and proposed the technique [15, 39]. Later, Arya et al. found no significant difference between the pyloroplasty and the control groups in their work, although the strength of these findings had limitations, because only seven RCTs were included in addition to 18 lower quality articles [16]. Other limitation were the small number of patients and the high heterogeneity of the definitions of outcomes in the enrolled studies.

In our meta-analysis, we provide the most precise and objective evidence available to date on the topic, due to the strict inclusion criteria of our work. Although the previous meta-analyses’ partial results confirm our findings, they overall favored intraoperative pyloroplasty or had a significant heterogeneity, therefore, we considered this rediscussion necessary. We used the latest protocol in our study, and only the latest randomized controlled studies were included to minimize the risk of bias.

As expected, **mortality** remained low in both arms in the individual enrolled studies, as esophagectomy is usually performed as an elective intervention. Based on our analysis, performing pyloric drainage combine with esophagectomy does not carry additional advantage compared to the omission of pyloric drainage (*p* = 0.642). This finding is also supported by the former systematic analyses (*p* = 0.86) [19]. Despite the fact, that mortality is the strongest investigated outcome, this might not present a clear view of the benefits and harms of such an intervention due to the formerly mentioned reasons. On the other hand, cancer-specific and overall survival should be investigated more thoroughly by future studies.

**Anastomotic leakage** is one of the most common complications of any kind of surgical resection. The additional operation can be associated with mortality and morbidity, therefore may influence this outcome. However, neither our analysis (*p* = 0.254) nor previous works (*p* = 0.12; *p* = 0.77) described such an effect or difference [16, 19].

Besides anastomotic leakage, another common complication of surgical procedures is **pulmonary morbidity**. Since none of the existing systematic reviews and meta-analyses found a significant difference (*p* = 0.214) (*p* = 0.31; *p* = 0.15) [16, 19], and Urschel et al. also have not reported any significant difference in risk of **fatal pulmonary aspiration** (*p* = 0.14), therefore pyloric drainage is not superior from this aspect [19].

Relative risk of **pyloric drainage complication** was reported (*p* = 0.33) [19], and we also have not found any benefit in terms of **vomiting** (*p* = 0.520). Although Urschel et al. found no evidence of biliary complications [19], but in the opinion of other authors, pyloric interventions may increase long-term **biliary reflux** (*p* = 0.069) and **reflux esophagitis** (*p* < 0.05), which can lead to poor quality of life, however, this long-term complication could not be analysed in this meta-analysis [15, 16].

Multiple methods can be used for examining the **gastric emptying time**; therefore, the results carry notable limitations. Arya et al. found only six comparative studies out of twenty-four, and they found no significant difference between the control and pyloroplasty groups in delayed gastric emptying (*p* = 0.22) [16]. Urschel et al. found that the 0.53 drainage versus no drainage ratio expressed a shorter emptying time in scintigraphy examination, but the semiquantitative analysis found no significance in gastric emptying [19]. However, we also found in seven RCTs shorter gastric emptying time, in the case of continuous data (*p* = 0.009), but in the dichotomic analysis, we found no significant difference based on four RCTs (*p* = 0.072) [18, 28–30].

Gaur et al. used delayed **gastric emptying and gastric outlet obstruction ratio**, which did not show a significant difference between the control and the pyloric drainage group (DGE/GOO of 8.1%, 13.2%) [15].

Although not clearly detailed, Urschel et al. found relative risk for **gastric outlet obstruction**, based on three RCTs [25, 28, 30]. (RR: 0.18, 95% CI, 0.03, 0.97; *p* = 0.046), but the semiquantitative analysis found no significant difference in **obstructive foregut symptoms** (−0.84) and **food intake** and **nutritional status** (+1.02) [19]. According to Gaur et al. pyloric drainage was ineffective in **dumping syndrome** [15].

The population and outcomes of our analysis are homogeneous. Due to the enrolment of randomized controlled trials the risk of bias is low. The TSA analysis is limited according to the recent guidelines, therefore they should be interpreted accordingly. In the case of some outcomes, the evidence is not conclusive, based on our TSA results. In these cases, further randomized clinical trials are needed. RCTs should focus on survival rather than mortality, as it provides a clearer picture of the most relevant outcome.

Limitations were caused by the rigorous inclusion criteria, which resulted in fewer clinical trials being available, thus a low number of patients. The other limitation is the date of the trials. The median year of origin of the articles was 1991, which carries a bias, due to development of the esophageal surgical procedures.

The proportion of available data of the demographic parameters about age, sex, stage, type of surgery, and resection were 66%, 55%, 22%, and 44%, respectively, which may affect the examined outcomes.

The population of the patients was overwhelmingly Asian while Europeans and South Africans were represented by only one article each [25, 30]. Thus, the application of these results to European and American populations is limited due to the seven Asian articles, in detail China, Hong Kong and India [24, 26–29, 31]. United Arab Emirates was also represented [18].

Another possible limitation is that certain outcomes were very heterogenic. The measurement of gastric emptying time used different methods and protocols (barium or isotope protocol), and some morbidity groups did not have clear clusters or artificial limit value was defined and the subjective sensations were also not clarified or not scanned at all.

## Conclusion

In conclusion, pyloroplasty during esophagectomy in elective surgery especially MIE has no substantial benefit based on our results, therefore it should be routinely omitted to reduce operating time. It does not affect mortality, anastomosis leakage, respiratory or another morbidity like vomiting, or reflux, but it has possible risks. Its effect on shorter gastric emptying time is unclear, and the beneficial consequence is questionable. If symptoms of gastric stasis occur, available postoperative pyloric drainage procedures are good options.

## Data Availability

The original contributions presented in the study are included in the article/Supplementary Material, further inquiries can be directed to the corresponding author.

## Supplementary material

SUPPLEMENTARY FIGURE S1 Anastomosis leakage, Forrest plot, OR: odds ratio, CI: confidence interval.

SUPPLEMENTARY FIGURE S2 Anastomosis leakage, trial sequential analysis (TSA) analysis.

SUPPLEMENTARY FIGURE S3 Respiratory morbidity, Forrest plot, OR: odds ratio, CI: confidence interval.

SUPPLEMENTARY FIGURE S4 Respiratory morbidity, trial sequential analysis (TSA) analysis.

SUPPLEMENTARY FIGURE S5 Vomiting, Forrest plot, OR: odds ratio, CI: confidence interval.

SUPPLEMENTARY FIGURE S6 Vomiting, trial sequential analysis (TSA) analysis.

SUPPLEMENTARY FIGURE S7 Gastric emptying time (GET) dichotomic data, trial sequential analysis (TSA) analysis.

SUPPLEMENTARY FIGURE S8 Gastric emptying time (GET) continuous data, Forrest plot, WMD: weighted mean difference, SD: standard deviation.

SUPPLEMENTARY FIGURE S9 Gastric emptying time (GET) continuous data, trial sequential analysis (TSA) analysis.
