# Supplementary figures and images for: Intraoperative pyloric drainage is unnecessary during esophagectomies: a meta-analysis and systematic review of randomized controlled trials
Source: Pathol Oncol Res. 2024 Aug 6;30:1611823. doi: 10.3389/pore.2024.1611823 (PMC11333203; doi:10.3389/pore.2024.1611823)

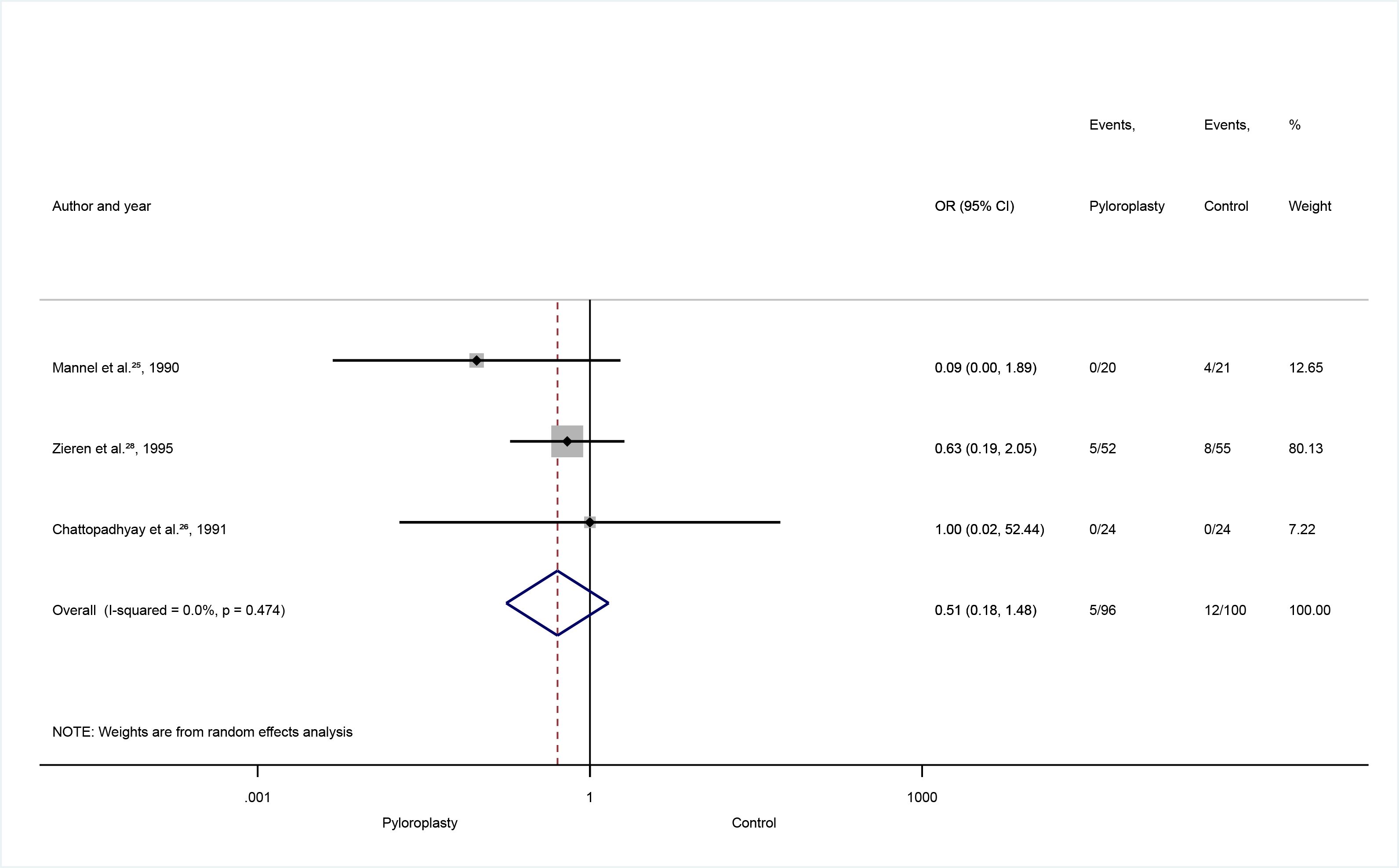

Supplement: Supplementary file 2 [file Image3.jpeg]

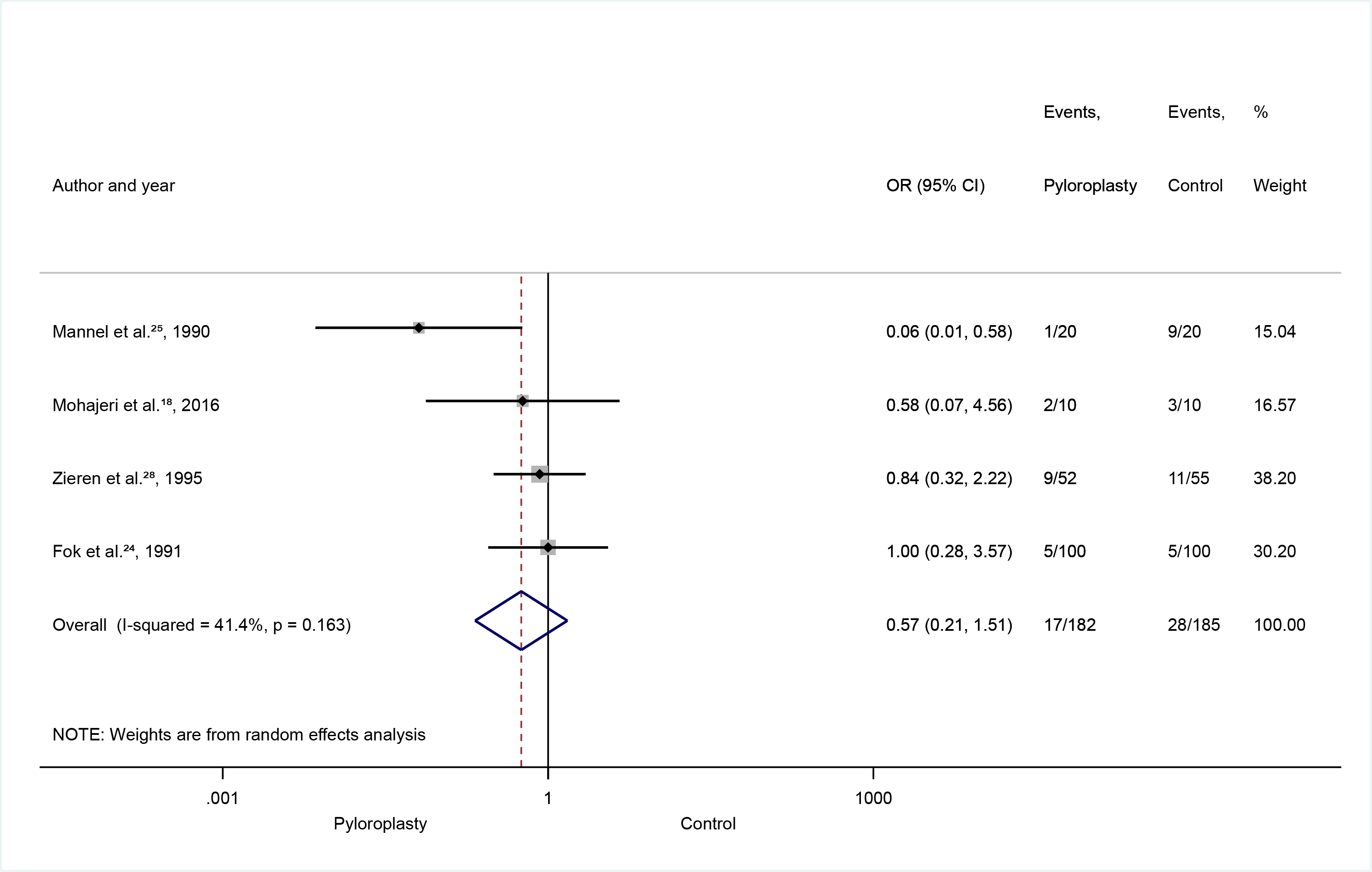

Supplement: Supplementary file 4 [file Image1.jpeg]

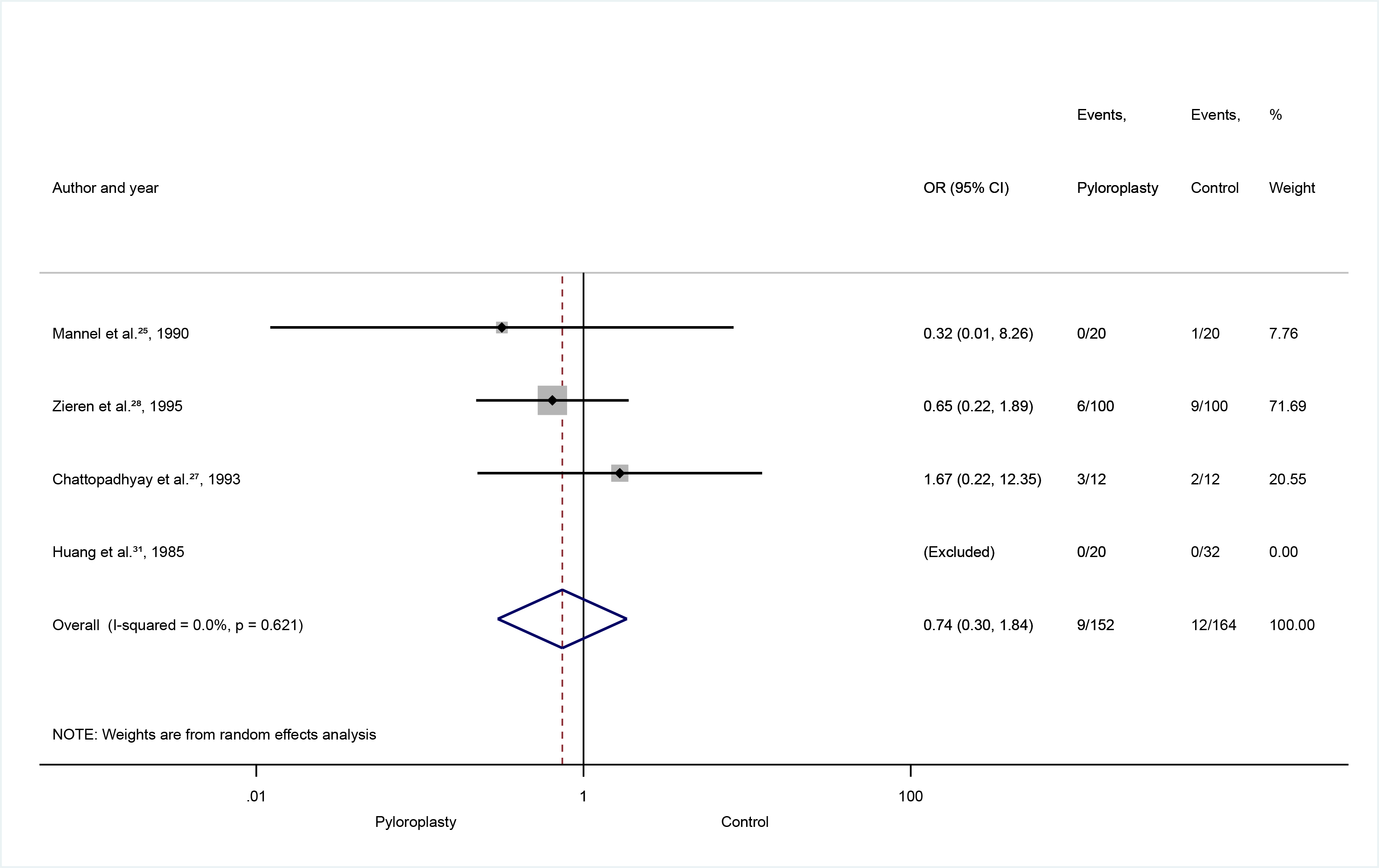

Supplement: Supplementary file 5 [file Image5.jpeg]

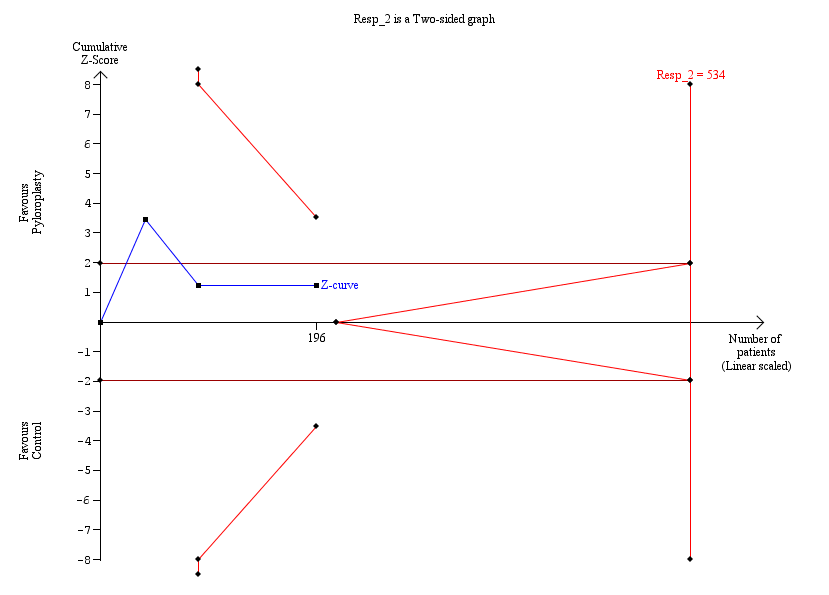

Supplement: Supplementary file 6 [file Image4.png]

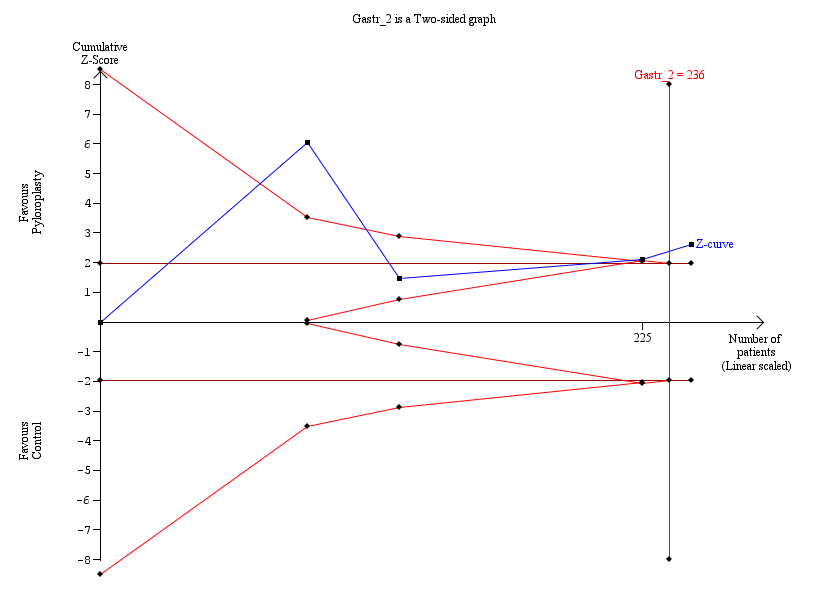

Supplement: Supplementary file 7 [file Image7.png]

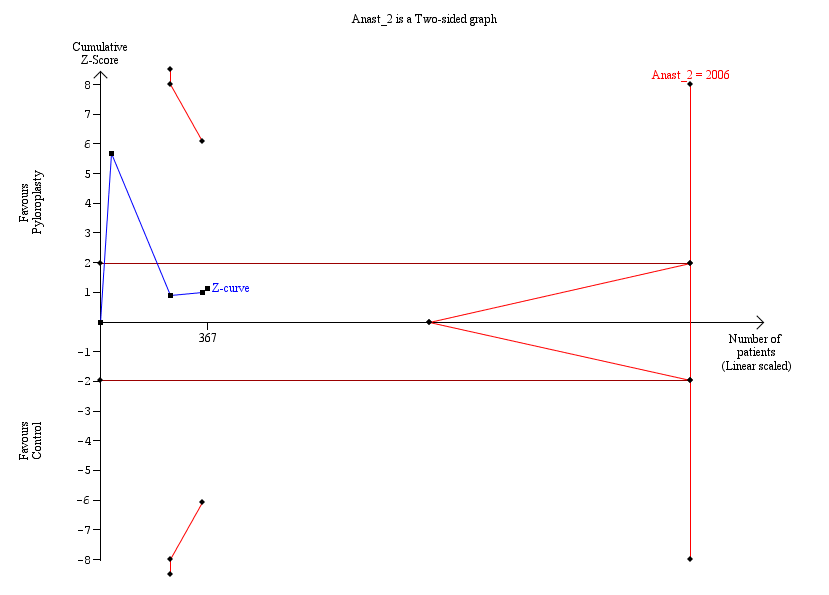

Supplement: Supplementary file 8 [file Image2.png]

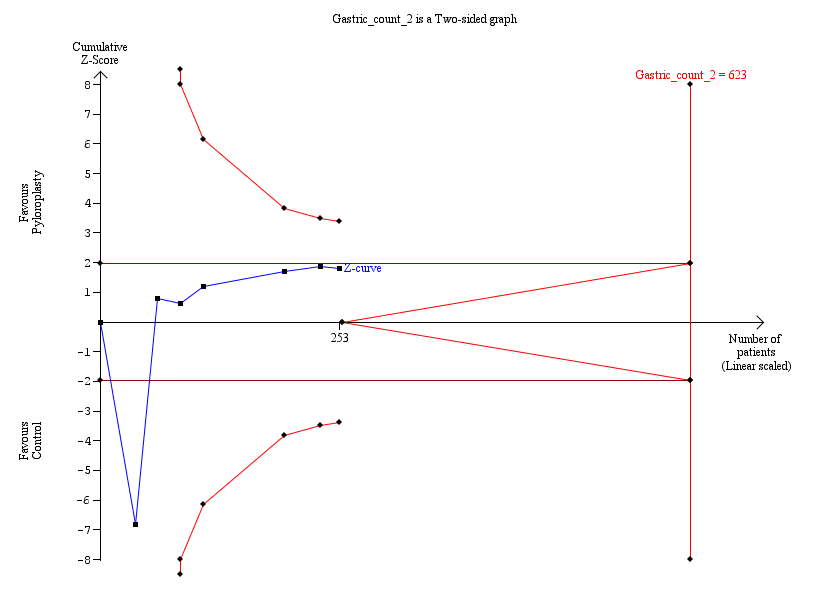

Supplement: Supplementary file 9 [file Image9.png]

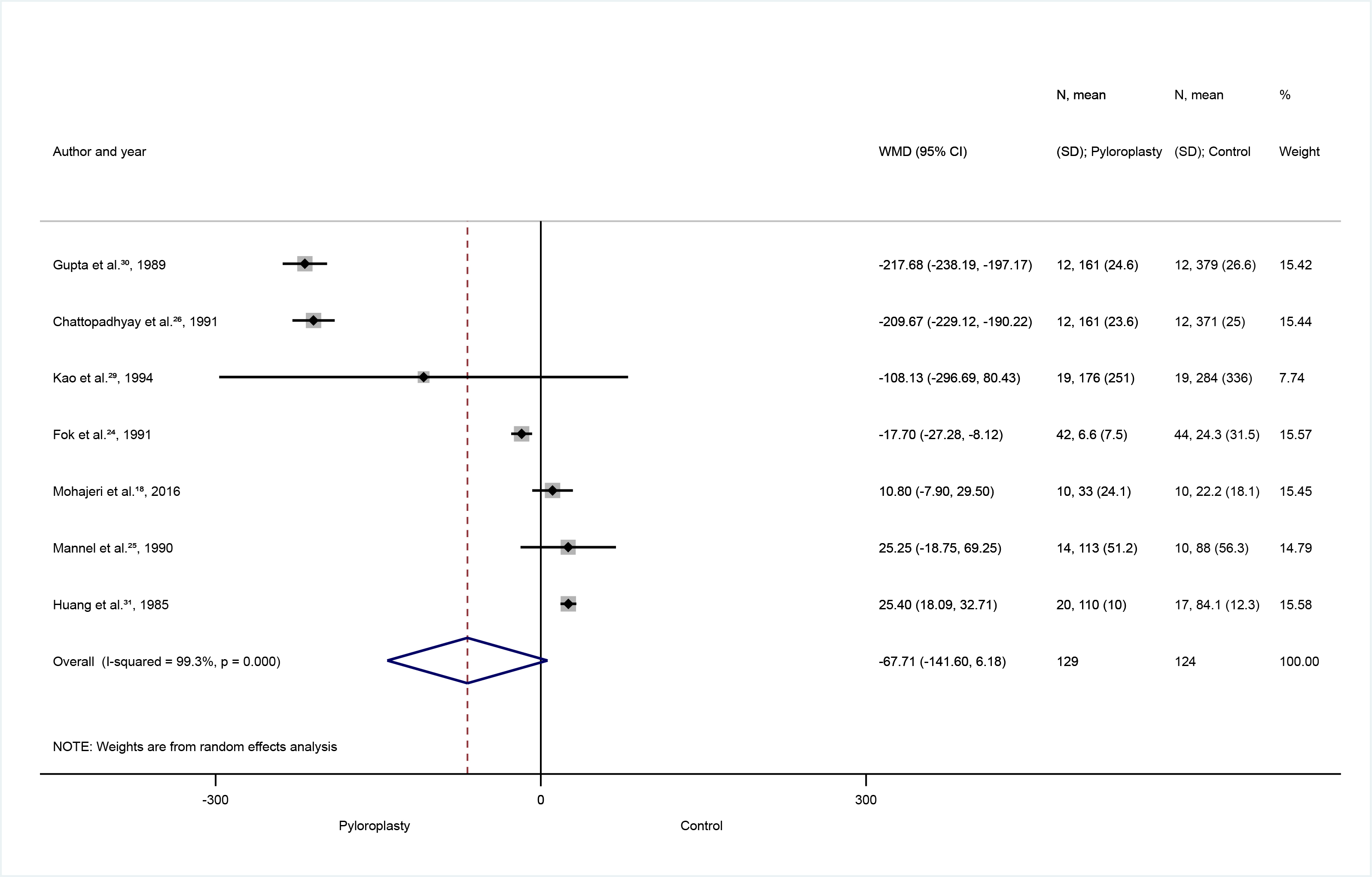

Supplement: Supplementary file 10 [file Image8.jpeg]

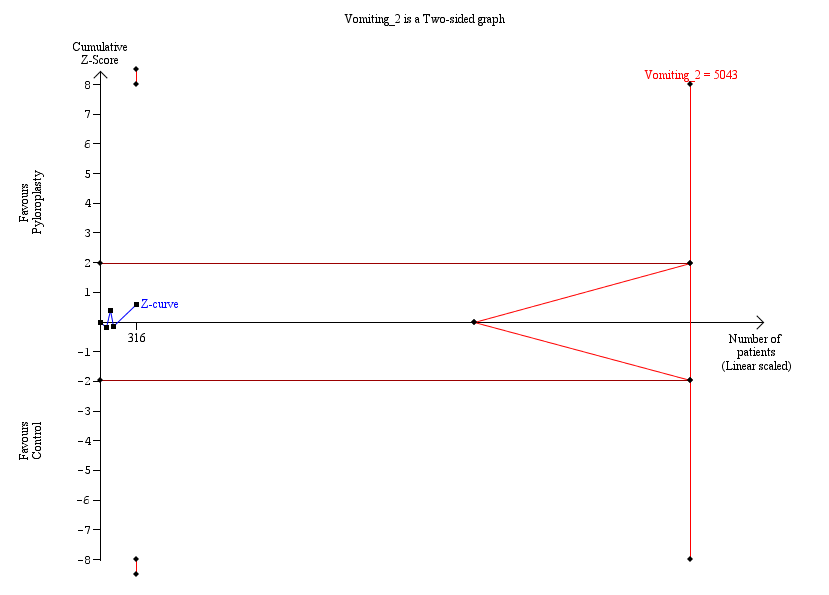

Supplement: Supplementary file 11 [file Image6.png]
