# Supplementary material for: Intraoperative pyloric drainage is unnecessary during esophagectomies: a meta-analysis and systematic review of randomized controlled trials
Source: Pathol Oncol Res. 2024 Aug 6;30:1611823. doi: 10.3389/pore.2024.1611823 (PMC11333203; doi:10.3389/pore.2024.1611823)
Supplement: Supplementary file 3 [file Table1.DOCX]

Supplementary Table 1: The results of the GRADE approach

| Outcomes | No of Participants (studies) | OR (95% CI) | I^2^ | Risk of Bias | Inconsistency | Imprecision | Effect of plausible residual confounding | Quality of the evidence |
| --- | --- | --- | --- | --- | --- | --- | --- | --- |
| Mortality | 347 (3 studies) | 0.85 (0.43, 1.69) | 0.0%; p=0.377 | - | - | - | ↓ | Moderate |
| Anastomosis leakage | 367 (4 studies) | 0.57 (0.21, 1.51) | 41.4%; p=0.163 | - | - | - | ↓ | Moderate |
| Respiratory Morbidity | 196 (3 studies) | 0.51 (0.18, 1.48) | 0.0%; p=0.474 | - | - | - | ↓ | Moderate |
| Vomiting | 316 (4 studies) | 0.74 (0.30, 1.84) | 0.0%; p=0.621 | - | - | - | ↓ | Moderate |
| Gastric Emptying Time (dichotomic) | 245 (4 studies) | 2.75 (1.28, 5.91) | 0.0%; p=0.487 | - | - | - | ↓ | Moderate |
| Gastric Emptying Time (continuous) | 253 (7 studies) | -67.71 (-141.60, 6.18) | 99.3%; p<0.001 | - | ↓ | ↓ | ↓ | Low |

Supplementary Table 1 is containing the results of the GRADE approach, which estimated the quality of the evidence. All of the outcomes have a low risk of bias, however, imprecision was decreased, due to the elderly article and the Asian predominance. The evidence of mortality, anastomosis leakage, respiratory morbidity, vomiting and gastric emptying time (dichotomic) were moderate, and gastric emptying time (continuous) was low, therefore, it should be interpreted accordingly. N0: number, CI: confidence interval.
